# It's about time: WHO and partners release programming strategies for postpartum family planning

**DOI:** 10.9745/GHSP-D-13-00156

**Published:** 2014-02-04

**Authors:** Mary Eluned Gaffield, Shannon Egan, Marleen Temmerman

**Affiliations:** a World Health Organization, Geneva, Switzerland; b Jhpiego, Maternal and Child Health Integrated Program (MCHIP), Washington, DC, USA

## Abstract

The postpartum period is a critical time to address high unmet family planning need and to reduce the risks of closely spaced pregnancies. Practical tools are included in the new resource for integrating postpartum family planning at points when women have frequent health system contact, including during antenatal care, labor and delivery, postnatal care, immunization, and child health care.

Since 2010, the World Health Organization (WHO) has been receiving an increasing number of requests from country programs for strategies to create or strengthen voluntary family planning programs for women in the first year after childbirth. During this extended postpartum period, 95% of women in low- and middle-income countries want to avoid a pregnancy within the next 2 years, but 70% are not using contraception.^1^

In collaboration with the Maternal and Child Health Integrated Program (MCHIP) of the U.S. Agency for International Development (USAID) and several other partners, WHO produced the “Statement for Collective Action for Postpartum Family Planning” to emphasize the importance of postpartum family planning (PPFP) and to offer general approaches for addressing unmet need and expanding the range of contraceptive options during the postpartum period.^2^ The global health community rallied in support of this obvious, but often overlooked, group of women in need of services. The Statement received official endorsements from additional donor governments, including Australia and the United Kingdom, and from family planning stakeholders, such as the United Nations Population Fund and the International Planned Parenthood Federation.

The 2012 London Summit on Family Planning coalesced renewed international commitment for family planning and highlighted PPFP's potential in accelerating progress toward Millennium Development Goals 4 and 5 (to reduce child mortality and improve maternal health, respectively). Some policy makers and program managers expressed uncertainty, however, about incorporating PPFP into their unique national and local contexts, especially in areas with cultural barriers to family planning for postpartum women and with low facility-based delivery coverage. (Facilities would provide entry points for integrating PPFP.) Others misunderstood or underestimated the risk of pregnancy in the postpartum period and believed that PPFP was either unnecessary or a less important investment than family planning for non-postpartum women.

Postpartum family planning has the potential to accelerate progress toward Millennium Development Goals 4 and 5.

To ensure that decisions about PPFP programs are informed by the best evidence and field-tested practices, WHO, with support from USAID and MCHIP and through contributions from a large community of PPFP technical experts, launched a highly anticipated companion piece to the Statement for Collective Action at the 2013 International Conference on Family Planning in Addis Ababa, Ethiopia. The resource, “Programming Strategies for Postpartum Family Planning,” provides a detailed reference for PPFP program design for a variety of cultural contexts (see supplementary material). It informs policy makers and program managers about the unique family planning needs of postpartum women, describes assessment methods to comprehensively identify PPFP programming opportunities, and presents illustrative strategies, complete with activities and measurable indicators, to integrate PPFP programs into multiple health system entry points.^3^

The document is specifically geared toward supporting program managers' efforts to:

- Mitigate missed PPFP opportunities across the continuum of care
- Organize health services to allow time for family planning counseling
- Maximize the availability of community-based care
- Expand the available range of family planning options and services

## POSTPARTUM WOMEN NEED FAMILY PLANNING, TOO

Although the postpartum period is clinically defined as the first 6 weeks following childbirth, **PPFP is the initiation of family planning services within the first 12 months following childbirth to prevent closely spaced and unintended pregnancies.** Pregnancies within the first 12 months after a birth—in other words, a birth-to-pregnancy interval of less than 12 months—are at highest risk for adverse health outcomes to the mother and child^4^; are much more likely to end in potentially unsafe induced abortion^5^; and are at elevated risk for stillbirth, preterm birth, low birth weight, and small size for gestational age.^4^ Closely spaced births are also correlated with increased likelihood of chronic undernourishment, stunted growth, and infant mortality.^6^ Because of these serious health risks, spacing pregnancies at least 2 years apart can avert an estimated 10% of infant deaths and 21% of deaths in children ages 1 to 4 globally.^4^

Spacing pregnancies at least 2 years apart can avert about 10% of infant deaths and 21% of deaths in children ages 1–4.

As a group, postpartum women have high unmet need for family planning, defined as the percentage of fecund and sexually active women who report not wanting any more children or wanting to delay the birth of their next child but are not using any method of contraception.^7^ One analysis of 27 low- and middle-income countries estimated that 65% of postpartum women had unmet need.^1^ A more recent analysis of data from 17 low- and middle-income countries found even higher unmet need estimates when women were asked about *prospective* need—that is, to express their fertility preferences looking into the future, instead of at the time of their previous pregnancy.^8^

An estimated 65% of postpartum women have unmet need for family planning.

## HOW POSTPARTUM WOMEN ARE DIFFERENT

Family planning services for postpartum women require unique physiological considerations. Postpartum women experience amenorrhea, or the absence of menses, for varying lengths of time, and their fertility can return before menses resumes, even when breastfeeding.^9^ PPFP programs also must understand the clinical safety standards applied to different contraceptive methods across the 12-month period following birth, taking the mother's breastfeeding status into special consideration.^3^ The Programming Strategies resource includes a tool for determining the appropriate method options throughout the first year postpartum, following the WHO Medical Eligibility Criteria for Contraceptive Use^10^ (Figure). These criteria are periodically reviewed to ensure that they are consistent with the latest evidence.

**FIGURE.**
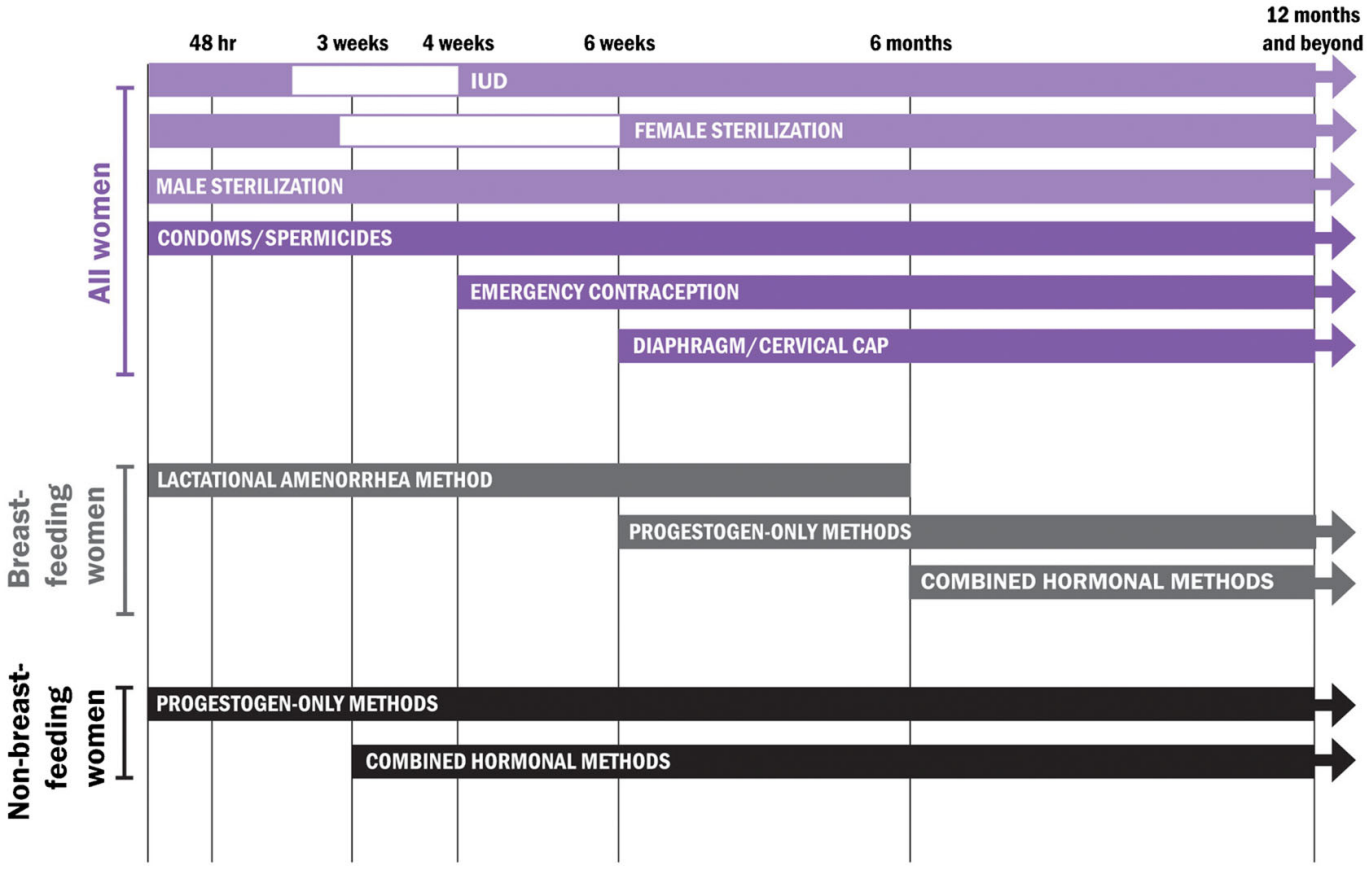
Use of Contraceptive Methods in the Postpartum Period, According to WHO's Medical Eligibility Criteria Source: World Health Organization^3^

## MITIGATING MISSED OPPORTUNITIES

Integrating PPFP services into antenatal care (ANC), labor and delivery, postnatal care (PNC), and well-child health visits allows programs to deliver family planning counseling and services during the points at which couples have the most frequent contact with the health care system, without substantial increases in staff or infrastructure.^2^^,^^9^ Effective PPFP use, however, relies on adaptation to established or developing health systems within each country, particularly in terms of the services that can be offered immediately after delivery, such as insertion of postpartum intrauterine devices (IUDs) post-delivery versus post-discharge. As a result, it can be difficult to effectively transfer models, even when they are employed successfully in other contexts. Nevertheless, careful planning and learning from challenges faced by PPFP programs in similar settings can overcome these obstacles.

Qualitative analysis on perceptions of effective access to and provision of PPFP services in Ethiopia and Kenya identified certain consistent programmatic requirements:

- **Tracking of postpartum contraceptive use** allows health workers, governments, and organizations to ensure the steady supply and distribution of contraceptive commodities, especially in rural areas.
- Availability of **high-quality, easy-to-understand informational materials** about PPFP and contraceptive options can help women and their families make informed choices.
- **Consistent health worker training and use of global PPFP best practices** ensure that service delivery is consistent with global standards for care.^11^

Government support is also a critical component of PPFP programming and can help ensure that voluntary family planning services are well-funded; are delivered consistently, safely, and effectively; and are technically and clinically sound. In cases where the government relies on or collaborates with funding sources from nongovernmental or private-sector partners, program managers may be able to advocate increased PPFP attention and support by leveraging these relationships.^3^

Government support for PPFP is critical to ensuring proper funding of programs.

## APPLYING PROGRAMMING STRATEGIES FOR POSTPARTUM FAMILY PLANNING

The new resource, “Programming Strategies for Postpartum Family Planning,”^3^ adapts the assessment questions identified by WHO's health systems framework,^12^ and it includes an additional element for determining community and sociocultural obstacles. It helps readers identify programmatic weaknesses related to the essential elements of any health system and provides examples of evidence-based interventions that program managers can adopt, depending on the findings in their assessments (Box). Although not intended to be exhaustive, these approaches should help direct attention toward interventions that strengthen service delivery, human resources, or financing. For example, if high rates of breastfeeding are noted, program planners can ensure that the Lactational Amenorrhea Method (LAM), and counseling on the transition from LAM to other effective contraceptive methods, are part of routine PNC and infant health care.

**BOX. Examples of PPFP Program Interventions**

**Table:** 

**Illustrative Assessment Findings**	**Potential PPFP Program Interventions**
• High unmet need for limiting future pregnancies • High percentage of births in facilities • Health system with district-level infrastructure for IUD and female sterilization services	**Facility-Based Intrapartum Services:** • Expand counseling and method mix to include long-acting reversible contraceptives (LARCs) and permanent methods (PMs), access, affordability, and choice. • Integrate immediate postpartum IUD insertion, postpartum tubal occlusion, and exclusive breastfeeding (EBF) within labor and delivery units and in postpartum maternity wards at facilities at the district or sub-district level, if appropriate.

• Low modern contraceptive prevalence • High use of traditional methods • Short birth intervals • High percentage of home births	**Community:** • Train community health workers to integrate community education and individual counseling about healthy timing and spacing of pregnancy (HTSP), EBF, and the Lactational Amenorrhea Method (LAM) with referral for other contraceptive methods as a routine part of care. • Promote early PNC visits for home births to provide essential newborn care and EBF/LAM. • Focus on LAM as a gateway method to using other modern contraceptives. • Discuss women's reproductive intentions for spacing or limiting, and provide information on contraceptive methods and where to get them. • Use community-based integrated maternal, newborn, child health, and family planning (MNCH/FP) services.

• Existence of insurance or other finance mechanisms, such as vouchers, for basic maternity services and PNC	**Financing:** • Bundle PPFP with the birthing package to ensure that all contraceptive methods are covered during the extended postpartum period.

• High breastfeeding rates • Successful routine immunization sessions at health centers	**PNC and Infant Care:** • Introduce LAM and transition to other contraceptive methods. • Add a dedicated family planning provider to existing routine immunization programs or link/refer women to the family planning unit at the clinic.
	
• High rates of staff rotation within and among facilities • Lack of skills and knowledge about PPFP among facility staff, including the provision of LARCs/PMs • Facilities lack available and trained staff to provide MNCH/FP services	**Strengthening Human Resources Capacity:** • Strengthen policies and practices to address staff development and retention to ensure that providers with family planning skills are available within ANC, labor and delivery, and PNC. • Introduce or strengthen a comprehensive reproductive health education curriculum that addresses safe motherhood, family planning, and neonatal and child health training issues. • Integrate concepts of PPFP within preservice education and ensure that PPFP and HTSP are well-covered in teaching curricula, practical training, and examinations. • Dispatch mobile outreach teams to facilities in the short term in order to provide services while building capacity of staff for the long term. • Focus on community-based PPFP interventions, including EBF, LAM, pills, injectables, and condoms, while addressing health worker and capacity needs at the facility level.

• High HIV prevalence and existence of PMTCT services	**Meeting the Needs of People Living With HIV/AIDS:** • Integrate PPFP with PMTCT services and promote use of EBF and LAM, as well as appropriate complementary feeding at 6 months, with transition to another effective contraceptive method.

Irrespective of the policy and programmatic choices made to capitalize on entry points across the continuum of care—ANC, labor and delivery, PNC, or infant health and immunization services—the document provides ample references to potential program goals, outcomes, strategies, activities, and indicators for each contact point. Of particular note, the document cites WHO's recently updated recommendations that women receive PNC for at least 24 hours after birth and additional PNC contacts on day 3, between days 7–14, and at 6 weeks after birth.^13^

To support programming decisions, several examples of programs with targeted PPFP components that have been implemented in multiple cultural contexts are provided in the resource. Brief descriptions of program efforts and observed results are included, and supplemental information on program indicators for monitoring and evaluation purposes is also outlined.

## TIME FOR ACTION ON PPFP

In July 2012, bold global goals were announced during the London Summit on Family Planning, where 32 countries and numerous donors, foundations, and organizations, including WHO, made global political commitments to expand access to voluntary family planning for 120 million more women and girls around the world by 2020 (the “FP2020 goals”). The momentum of the Summit and the commitments that followed have not only renewed global attention on family planning as a key element in achieving development targets but also underscored the importance of coordinated action among various ministries and government entities, private and public sectors, stakeholders, and donors. The development of strong programs that can effectively meet the family planning needs of postpartum women while maintaining high-quality counseling and service delivery requires multisector collaboration among stakeholders at a variety of programmatic levels—ranging from facility directors, community health workers, and national program managers to private donors and international nongovernmental organizations. The careful coordination of these entities advances program practices that are based on sound sexual and reproductive health policies and standards of care and establishes robust, sustainable programs that have the ability to withstand and adjust to contextual changes. “Programming Strategies for Postpartum Family Planning” makes it easier to develop family planning programs by providing practical assessment tools for each step of the process, helping countries to identify the right solutions for their problems and employ the best strategies in their unique situations.

Comprehensive PPFP programming requires many types of health worker involvement at each point of interaction along the prenatal-to-postpartum continuum of care.

### Research Agenda Essential for PPFP

Monitoring and evaluation of program activities and outcomes—an often overlooked and underfunded aspect of program design—must be an essential component of strengthening programs. Improved measures and evaluations of an intervention's feasibility, cultural and contextual acceptability, and cost-effectiveness are critical to ensuring that PPFP programs are implemented rationally and expeditiously. Such measurements inform policy and ongoing learning, enable improvements in service delivery models, and allow programs to maximize benefit while minimizing known challenges. “Programming Strategies for Postpartum Family Planning” underscores the importance of holistic monitoring and evaluation and recommends valuable data collection methods and analytic metrics that are cost-effective and evidence-based and that address the most demanding programmatic obstacles.

Interest in family planning has also received substantial attention from the research community. Respondents to a recent WHO research priority setting exercise scored implementation issues related to PPFP among the highest of family planning research priorities.^14^ The expert group of 180 stakeholders identified PPFP service integration mechanisms and the development of effective strategies to overcome barriers to contraceptive uptake during the postpartum period as the second and fourth highest research priorities among a list of 47 topics. A new review of 34 PPFP programs confirms the difficulty of assessing program effectiveness without rigorous research designs and outlines the plethora of PPFP interventions that have not been studied systematically.^15^ There is an urgent need to scientifically investigate why certain PPFP interventions work—and why others do not—and why some integration opportunities that seem obviously beneficial, such as those at immunization visits, have shown weaker results than expected. By documenting and analyzing early findings of different PPFP models,^16^ and by following the research practices recommended by “Programming Strategies for Postpartum Family Planning,” we will be able to enhance best practices and develop stronger recommendations on how to direct human and financial resources.

## CONCLUSION

Given the few short years for us to reach FP2020 goals, we must take immediate action to encourage country programs to offer the family planning services that postpartum women want and deserve. The release of “Programming Strategies for Postpartum Family Planning” will help propel these programs in the right direction and enable the international community to reach this large and important population.
